# Knowledge and use of edible mushrooms in two municipalities of the Sierra Tarahumara, Chihuahua, Mexico

**DOI:** 10.1186/1746-4269-10-67

**Published:** 2014-09-17

**Authors:** Miroslava Quiñónez-Martínez, Felipe Ruan-Soto, Ivonne Estela Aguilar-Moreno, Fortunato Garza-Ocañas, Toutcha Lebgue-Keleng, Pablo Antonio Lavín-Murcio, Irma Delia Enríquez-Anchondo

**Affiliations:** Instituto de Ciencias Biomédicas, Universidad Autónoma de Ciudad Juárez, Ciudad Juárez, Chihuahua, México; Facultad de Ciencias Biológicas, Universidad de Ciencias y Artes de Chiapas, Tuxtla Gutiérrez, Chiapas México; Facultad de Ciencias Forestales, Universidad Autónoma de Nuevo León, Linares, Nuevo León México; Facultad de Zootecnia, Universidad Autónoma de Chihuahua, Chihuahua, Chihuahua, México

**Keywords:** Wild edible mushrooms, Mestizos, Raramuris, Forest, Chihuahua

## Abstract

**Background:**

The Sierra Madre Occidental of Chihuahua in Northern Mexico is inhabited by indigenous Raramuris, mestizos, and other ethnic groups. The territory consists of canyons and ravines with pine, oak and pine-oak forests in the higher plateaus. A great diversity of potentially edible mushrooms is found in forests of the Municipalities of Bocoyna and Urique. Their residents are the only consumers of wild mushrooms in the Northern Mexico; they have a long tradition of collecting and eating these during the “rainy season.” However, despite the wide diversity of edible mushrooms that grow in these areas, residents have a selective preference. This paper aims to record evidence of the knowledge and use of wild potentially edible mushroom species by inhabitants of towns in the Sierra Tarahumara of Chihuahua, Mexico.

**Method:**

Using a semi-structured technique, we surveyed 197 habitants from seven locations in Urique, Bocoyna, and the Cusarare area from 2010 to 2012. Known fungi, local nomenclature, species consumed, preparation methods, appreciation of taste, forms of preservation, criteria for differentiating toxic and edible fungi, other uses, economic aspects, and traditional teaching were recorded. To identify the recognized species, photographic stimuli of 22 local edible species and two toxic species were used.

**Results:**

The respondents reported preference for five species: *Amanita rubescens, Agaricus campestris, Ustilago maydis*, *Hypomyces lactifluorum,* and the *Amanita caesarea* complex. No apparent differences were found between ethnic groups in terms of preference, although mestizos used other species in Bocoyna (*Boletus edulis* and *B. pinophilus)*. Some different uses of fungi are recognized by respondents, i.e. home decorations, medicine, as food in breeding rams, etc.

**Conclusion:**

The studied population shows a great appreciation towards five species, mainly the *A. caesarea* complex, and an apparent lack of knowledge of nearly 20 species which are used as food in other areas of Mexico. There are no apparent differences among Sierra inhabitants in terms of gender, occupation, or language regarding the recognition and consumption of species. The rejection of certain species is due mainly to fear of poisoning and the traditional selective teaching of families in the mountain communities of the Sierra Tarahumara.

**Electronic supplementary material:**

The online version of this article (doi:10.1186/1746-4269-10-67) contains supplementary material, which is available to authorized users.

## Background

**Figure 1:**
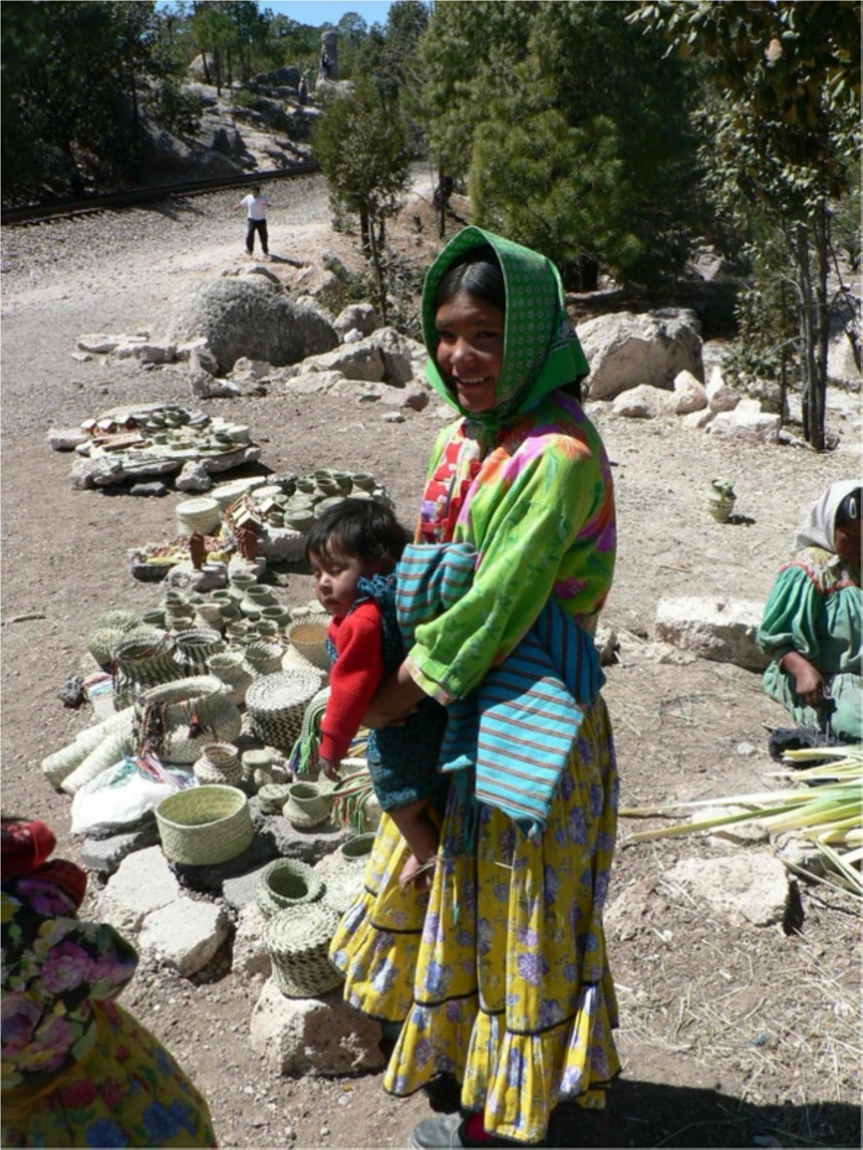
**Raramuri girl selling palm crafts in the Sierra Tarahumara.**

Chihuahua is the largest state in Mexico. It comprises 12.6% of the country’s area [1]. The southwestern portion of the Sierra Madre Occidental in this state is known as Sierra Tarahumara for it is occupied by an ethnic group known as the Raramuri or Tarahumara (Figure 1), which means “light footed people”. Even though the Raramuris represent the largest group in the area, other ethnic groups such as the Tepehuanos or Odame; the Guarojios, or Guarijo; the Pimas; and numerous other mountain mestizos called “Chabochi” or “Yori” [2–4] inhabit the region, and all together they comprise a population of approximately 270,000 people [1]. The main activities that sustain them are: forest logging, mining, trade, small-scale seasonal agriculture, agro-pastoralism, and artisanal production [5], where the later three meet the needs of Raramuris [6].

**Figure 2:**
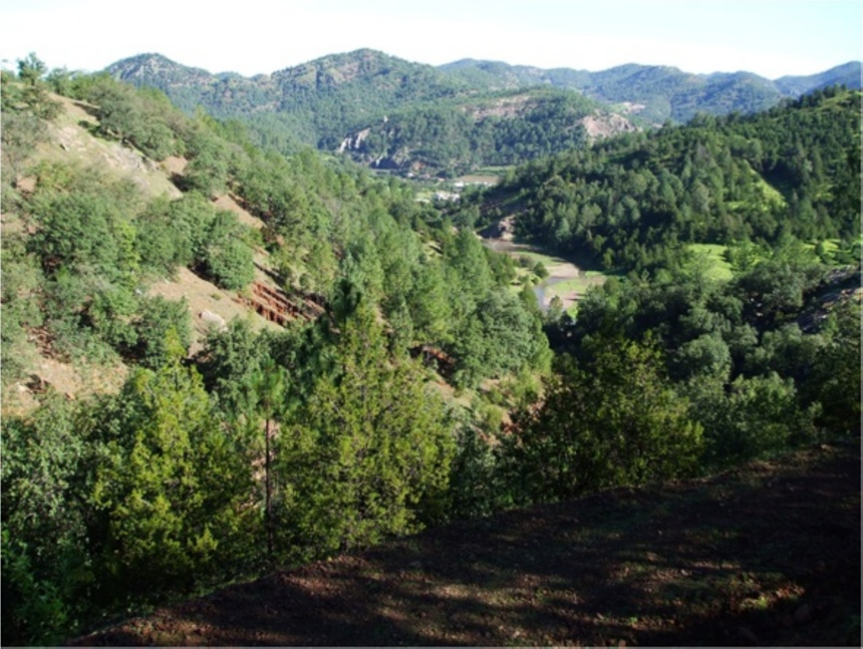
**Landscape forest vegetation of the Sierra Tarahumara.**

The territory that forms the Sierra Tarahumara consists of canyons and ravines where plant communities in the higher plateaus are pine, oak, and pine-oak forests [7] (Figure 2). Total annual precipitation ranges from 600 to 1200 mm, with an annual average of 705 mm. There is a well-defined season during the months of July to September which accounts for 68% of total precipitation [8]. Those forests harbor a variety of macroscopic fungi [9, 10], and some of them recognized by the villagers as food and are consumed during the rainy season [11].

Wild mushrooms are a non-timber forest resource valued by mycophilic human populations around the world [12–14]; their use has been recorded in many countries, and they are exploited commercially as food or medicine [15, 16]. Recent studies (within the last 10 years) are scarce in the state of Chihuahua [17–20]. Moreno et al. [21] mentioned that there are about 450 species studied so far, and this number is considered low due to the magnitude of the ecological diversity and size of the region. This positions the Sierra Tarahumara as a region with a great richness and diversity of fungi within an important and rich culture inhabited by indigenous and mestizo people. However, due to the orographic conditions, which make it difficult to reach, plus the isolated way of living of the Raramuris, this region is one of the least studied as far as mycocultural patrimony is concerned [3]. There are only a few studies that document ethnomycological data in the region [22–24]. In 2002, Moreno [25] conducted a study, specifically in two Raramuri populations (Panalachi and Tónachi) isolated from the influence of the mestizos, reporting 22 taxa with local ethnomycological importance. In a study conducted in the coniferous forest of the Sierra Tarahumara, Quiñonez et al. [26] reported a list of 50 wild mushrooms considered by the literature as potentially edible [27–29], including the results of a pilot survey on the potential use of some species by 50 people from the town of San Juanito, Chihuahua, highlighting the *Amanita caesarea* complex as the most consumed mushroom [26]. Other studies in different parts of Mexico showed that wild mushroom consumption is not standard nor generalized in the country, meaning that people tend to consider them to a lesser extent as a reliable food source [30–32]. Fear of poisoning and potential mortality associated with mushroom consumption [11, 31, 32] could be the possible causes linked to the low use of the fungal resources in the area, a fact that was highlighted by different researchers in the country [33].

Therefore, the objective of this study was to register and systematize the knowledge and use of the edible mushrooms in some parts of the Sierra Taharumara and hence contribute to the documentation of a biocultural patrimony in the least studied regions of Mexico.

## Methods

### Study area

**Figure 3:**
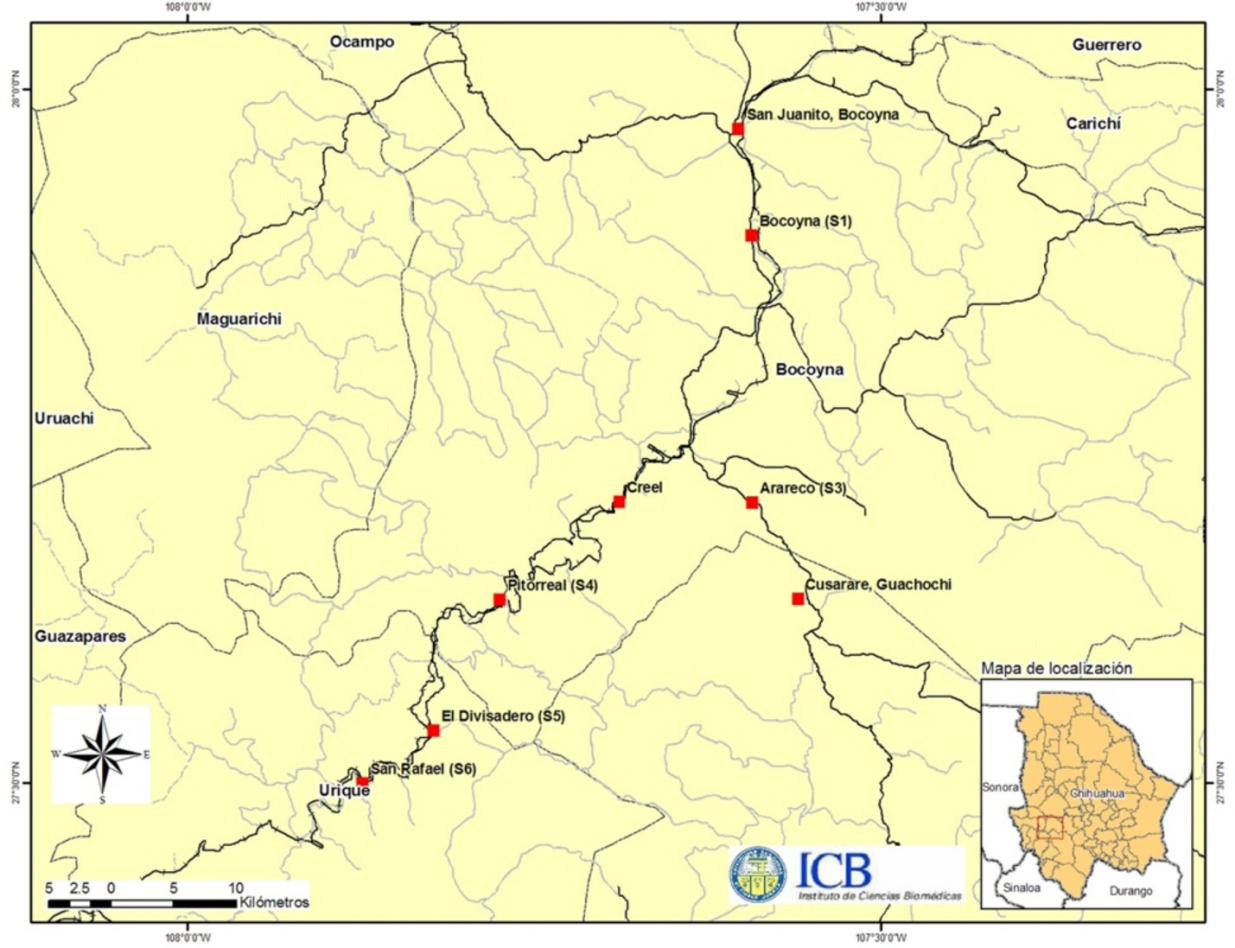
**Location of the study area.**

The study was conducted in the localities of: San Juanito, Bocoyna, Arareco, and Creel in the municipality of Bocoyna; Pitorreal, El Divisadero, and San Rafael belonging to the municipality of Urique; and at the Cusarare waterfall in the municipality of Guachochi in the state of Chihuahua (Figure 3).

San Juanito, Bocoyna, Arareco, and Creel are located in the upper part of the Sierra Madre Occidental at 220 km southwest of the city of Chihuahua, at 27° 30’ and 28° 30’ latitude north and between 107° 00’ and 108° 00’ longitude west. They have an average altitude of 2,350 meters with a maximum of 3,400 m. In general, the vegetation communities are made up of pine forest (P), pine-oak forest (Pq), oak-pine forest (Qp) and chaparral. There are areas with steep slopes with the presence of shallow soils belonging to the groups of Ferozems and Lithosols, characterized by a thin horizon layer containing little organic matter (humus) and some areas with deep soils used as agricultural lands [8]. The municipality of Bocoyna has a total of 505 villages with 28,766 inhabitants. Two urban areas are considered to have high number of inhabitants: San Juanito has 10,535 inhabitants, of which 152 are speakers of indigenous languages and Creel has 5,026 inhabitants with 350 Raramuris (the rest considered mestizos). The main activities of the inhabitants are local commerce, forestry, and tourism [34, 35]. Pitorreal, El Divisadero, and San Rafael in the municipality of Urique have an average altitude of 2,120 meters, with a maximum of 2,299 m and are located geographically between 27° 29’ and 27° 37’ latitude north, and 107° 52’ to 107° 46’ longitude west. The higher vegetation layer of these forests is formed mainly by the pine species: *Pinus arizonica*, *P. engelmannii*, *P. durangensis*, and *P. leiophylla* associated with oak species, mainly *Quercus arizonica*, *Q. chihuahuensis*, *Q. jonesii*, *Q. mcvaughii*, *Q. crassifolia*, *Q. depressipes*, *Q. durifolia*, and *Q. hypoleucoides*. Their combination forms plant communities of pine-oak forest (Pq) and oak-pine forest (Qp) [36, 37]. According to the National Institute of Statistics and Geography (INEGI), Pitorreal has a population of 14 inhabitants, El Divisadero11, and San Rafael 2,160 and the latter considered one the most important towns for being the most populous of the Municipality of Urique. The number of indigenous speakers in this location is 369. The main economic activities are forestry, tourism, and commerce [35].

The Cusarare waterfall, in the municipality of Guachochi, is located 25 km southeast of Creel, Chihuahua. It has a fall of 30 m during the months of July to October, and the surrounding vegetation is made up of pine forest. This waterfall is one of the main tourist attractions and sources of income for some residents in the area, mainly the ethnic Raramuri group [38]. The area of Cusarare has 106 inhabitants, and 19 of them are native speakers [35]. All sites are characterized by the sale of handicrafts made by both indigenous and mestizo people, which they sell to domestic and foreign tourists visiting the tourist sites and towns in Bocoyna and Urique, mainly El Divisadero, Barrancas, and Creel.

### Ethnomycological study

A study on fungal consumption and use by the inhabitants of several communities was conducted from 2010 to 2012. Before starting work, permission was requested of the civil authorities of the municipalities in order to carry out the study. In addition, each person interviewed was asked verbally for his/her consent and was informed that the data would be used for the present study. Semi-structured interviews as proposed by Bernard [39] were given to 197 people in the study area (Table 1).

**Table 1:** **Localities of the interviewed people of the Sierra Tarahumara**

Localities	n (%)
San Juanito	65 (33)
Creel	48 (24)
San Rafael	30 (15)
El Divisadero	14 (7)
Pitorreal	11 (6)
Bocoyna	11 (6)
Cusarare	11 (6)
Arareco	7 (4)

All respondents were asked if they were willing to be interviewed about their knowledge and use of wild mushrooms growing in the region where they live, informing them previously of the objectives of the study and that if they decided to participate, their answers would be used for a scientific publication. Only those who gave their express informed consent were subsequently interviewed, respecting the decision of those who refused to participate in the investigation. The study was approved by the Research Ethics Committee of University Autonomy of Ciudad Juárez(CBE.ICB/20.08-14).

**Figure 4:**
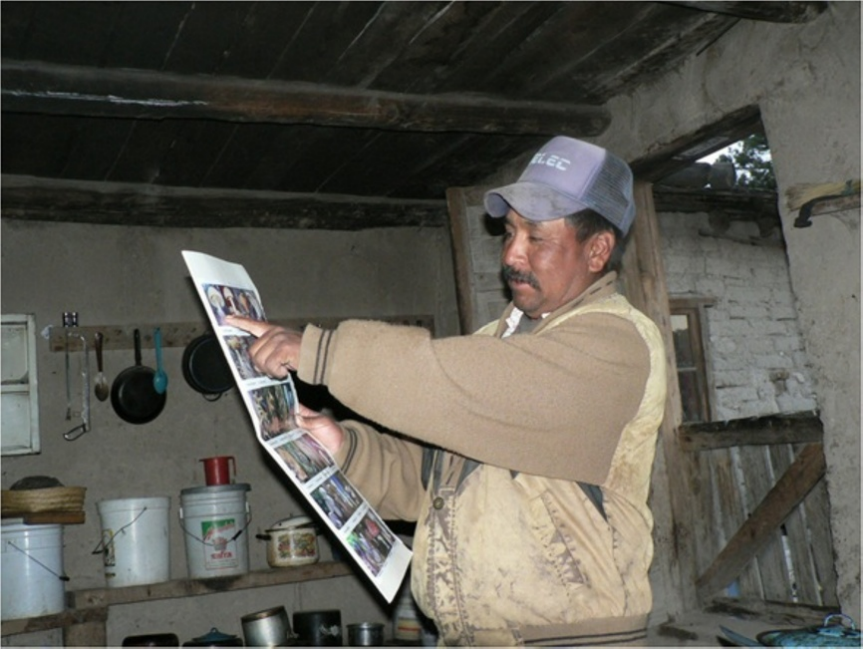
**A resident of the Sierra Tarahumara indicating the photograph of his mushroom of choice.**

**Figure 5:**
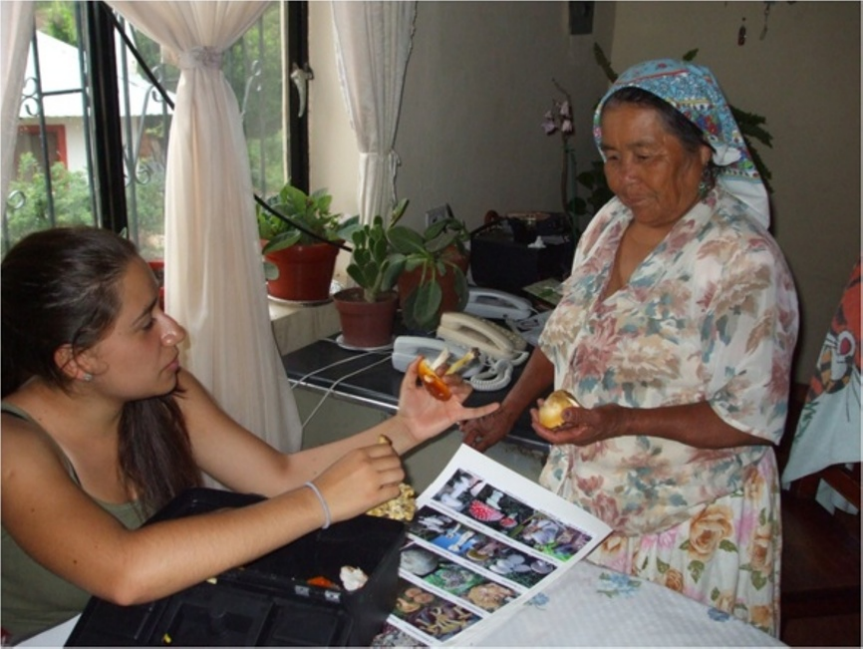
**Interview with a housewife showing fresh mushrooms and wild mushrooms of the Sierra Tarahumara.**

From each interviewed person, the following information was obtained: known fungi, local nomenclature, species consumed, preparation methods, appreciation of taste, forms of preservation, criteria for differentiating toxic and edible fungi, other uses, economic aspects, and ways of knowledge transmission. Also included were questions on sociodemographic information such as age, gender, occupation and ethnicity (Raramuris or mestizos). For the semi-structured interviews, pre-established formats were used (Additional file 1: Annex). To identify the species recognized by respondents, photographic stimuli of 22 edible species that commonly grow in the Sierra Tarahumara plus two toxic species were used: 1. *Amanita caesarea* complex; 2. *A. rubescens* Pers; 3. *Hypomyces lactifluorum* Schwein. Tul & C.Tul; 4*. Russula brevipes* Peck; 5. *Boletus chrysenteron* Bull; 6. *Laccaria laccata* (Scop.) Cooke; 7*. Boletus pinophilus* Pilat & Dermek; 8. *Boletus edulis* Bull; 9. *Cantharellus cibarius* Fr; 10*. Lactarius deliciosus* (L.) Gray; 11. *Auricularia polytricha* (Mont.) Sacc; 12. *Coprinus comatus* O. F. (Müll.) Pers; 13. *Ramaria* aff. *flava* Quél; 14. *Morchella vulgaris* (Pers.) Boudier; 15. *Hericium erinaceus* (Bull.) Persoon; 16*. Lactarius indigo* (Schwein.) Fr; 17. *Agaricus campestris* L.: Fr.; 18. *Boletellus russellii* (Frost) Gilbert; 19. *Helvella crispa* Bull; 20. *Schizophyllum commune* Fr; 21. *Ustilago maydis* (DC.) Corda; 22. *Helvella lacunosa* Afzel; 23. *Amanita muscaria* (L:Fr.) Lam, and 24. *Amanita virosa* (Fr.) Bertill (Figures 4 and 5). These species were selected for references of records and abundant growth and for being common in these forest soils [10, 36]. For the stimuli, the technique proposed by Thomas [40] was taken into account. Besides these photographic stimuli, in so far as possible, fresh mushrooms were used for correlation with the taxonomic fungi mentioned in the interviews. The collected samples were described in terms of macroscopic characteristics and were photographed and classified according to the [41] proposed by Cifuentes et al. Subsequently the specimens were reviewed microscopically following conventional mycological techniques [42]. Specialized taxonomic keys were used to determine the different specimens. Finally, these were deposited in the Biodiversity Herbarium of the Institute of Biomedical Sciences of the Autonomous University of Ciudad Juarez. Qualitative analysis of the information obtained was performed through constant comparison of categories according to the analysis proposed by Sandoval [43]. The names of fungi in Raramuri were written and verified by an expert in the Raramuri language working in the Language Center of the Autonomous University of Ciudad Juarez.

## Results and discussion

### Overview of the interviewed population

Of the 197 respondents, 30% were retailers (mainly groceries, fruit, vegetables, meats, and basic household products) and all of them were mestizo, 19.2% were students (mainly from the elementary and secondary level), 9.13% were housewives, 3.5% were professionals from fields such as medicine, nursing, nutrition, law enforcement, and accounting (with one to two respondents per each field); 29.4% were from diverse occupations such as farmers, artisans, mill workers, auto mechanics, construction workers, drivers, servants (Table 2); and the rest did not want to disclose their occupation (7.61%). From this population, 146 (74%) people were mestizo or “chabochi” (a term given to people who do not belong to the ethnic Raramuri), and 51 (26%) were Raramuris. Regarding gender classification, 56% of the surveyed sample was female (n = 110) and 44% was male (n = 87). In addition, ages ranged from 8 to 60 years old, although most of the respondents were between from 25 and 45 years old.

**Table 2:** **Occupation, gender, and ethnicity of the interviewed population (n = 197)**

Activities	n (%)	Gender	n (%)	Ethnic group	n (%)
Retailers	61 (30.9)	Female	110 (56)	Mestizo	146 (74)
Students	38 (19.2)	Male	87 (44)	Raramuri	51 (26)
Diverse occupations	58 (29.4)				
Professionals	7 (3.55)				
Housewives	18 (9.13)				
Not disclosed	15 (7.61)				

### Recognized species

The results of the interviews from the inhabitants of the eight villages of the municipalities of Bocoyna, Urique, and Guachochi showed that the 24 species were known to the interviewees. *Amanita caesarea* complex, *A. muscaria,* and *A. rubescens* turned out to be the species most people recognized, being identified by 92%, 84%, and 78%, respectively, of the total population surveyed (Table 3). More than 50 inhabitants recognized five species: *A. campestris,* and *U. maydis* plus the above three; 30 to 40 people recognized *Boletus edulis*, *B. pinophilus* and *A. virosa*; and the remaining 16 species were recognized by less than 24 of the inhabitants interviewed (Table 3). No apparent differences were found among people of different gender or speakers of different languages. Species recognition ranged from one to six among women, men, mestizo, and Raramuri people. Only one mestizo, in the town of Creel, recognized 23 species.

**Table 3:** **Frequency of recognition and use of species of edible mushrooms (N = 197 people)**

Species	n (knowledge)	n (use)
*Amanita caesarea* complex	182	164
*Amanita muscaria* (L:Fr.) Lam	165	0
*Amanita rubescens* Pers	153	89
*Ustilago maydis* (DC.) Corda	63	34
*Agaricus campestris* L.: Fr.	57	30
*Boletus pinophilus* L.: Fr.	40	14
*Amanita virosa* (Fr.) Bertill	32	0
*Boletus edulis* Bull	30	10
*Cantharellus cibarius* Fr.	23	6
*Russula brevipes* Peck	18	3
*Hypomyces lactifluorum* Schwein. Tul & C.Tul	17	5
*Lactarius deliciosus* (L.) Gray	14	5
*Hericium erinaceus* (Bull.) Persoon	14	0
*Schizophyllum commune* Fr.	14	1
*Auricularia polytricha* (Mont.) Sacc.	12	1
*Ramaria* aff. *flava* Quél.	10	3
*Laccaria laccata* (Scop.) Cooke	9	1
*Boletus chrysenteron* Bull.	8	0
*Lactarius indigo* (Schwein.) Fr.	8	0
*Boletellus russellii* (Frost) Gilbert	8	0
*Coprinus comatus* O. F. (Müll.) Pers	7	1
*Helvella lacunosa* Afzel	7	0
*Helvella crispa* Bull	6	3
*Morchella vulgaris* (Pers.) Boudier	4	0

### Frequency of consumption of edible species

Of the 22 species used as stimuli, the interviewed population consumed only 16; the remaining six species, although edibility is recognized, were not consumed. *Amanita caesarea* complex was consumed by 83% of respondents, and *A. rubescens* by 45%. In lower proportion *U. maydis* and *A. campestris* were consumed by 17% and 15%, respectively, and 10 to 14% of the respondents liked *B. pinophilus* and *B. edulis* (mostly by people living in the town of San Juanito) these were identified by their spongy texture known as “Boletus”, sponge, or “father’s cap”. Only one man, 72 years of age, and living in the town of San Juanito mentioned that he consumed seven species of fungi, and 36 of the interviewed people consumed only one species, specifically *A. caesarea* complex. Nine of the respondents did not consume fungi; mainly for fear of poisoning because they were aware of deaths of people caused by fungi elsewhere, or simply they did not like their taste or appearance. Most of these people were from San Rafael, Urique (Table 3). Men as well as women consumed on average, two species of mushrooms (with five to seven as the maximum and one as the minimum). This same pattern was found with the mestizos and Raramuris, and the same applied within their different occupations. These results indicated that knowledge of and use made by most mestizos and Raramuris of the middle and top of the Sierra Tarahumara were related to two particular species: *A. rubescens* and *A. caesarea* complex and, to a lesser extent, *A. campestris*. These results coincide in large part with the work of Moreno [25], who performed a similar study in other towns in the Sierra Tarahumara, but specifically with indigenous Raramuri, and concluded that these species were known and consumed. So, regardless of ethnicity, by far these species were the most appreciated in the forests of Chihuahua, with differences in terminology or common names (Table 4). However, this is not the case in all the populations where mushrooms are consumed in Mexico. Some ethnic groups were more similar in their use of certain edible species according to the geographical regions and vegetation types they inhabited [25, 44]. For example, studies by Garibay et al. [45] referred to *Cantharellus cibarius*, as the most frequently consumed species by the Zapotec people from Oaxaca, Mexico, but also they mentioned the complex of *A. caesarea* as the most economically important. Grajales-Vasquez et al. [46] reported the preference of edible species in the town of Independence in the state of Chiapas, which included species of such genera as: *Pleurotus, Polyporus*, and *Favolus,* (commonly found in tropical areas), but they also recorded *A. caesarea* complex and *C. cibarius* for the higher temperate zones. In an ethnomycology study in two communities in the Lacandon rainforest in Chiapas, Mexico, Ruan-Soto et al. [47] mentioned the use of 10 species of fungi, including nine lignicolous that, according to the perception of respondents, grow on wood and are edible. In this study, *Schizophyllum commune* was the most known and consumed by the people interviewed in both communities, and it may be considered by the authors as possibly the most prized edible mushroom in the tropical areas of the world [47]. This fungus commonly grows on the trunks of the oaks in the middle and lower parts of the Sierra Tarahumara [20]. In our study, only one person mentioned it as edible, and in Moreno’s study [25], it was not mentioned as being used by the Raramuris. More than 50 species of potentially edible wild fungi grow in these forests [9, 10, 21, 26, 48]. Within the few species that are consumed, *A. rubescens* was considered the second option during the month of August, and those who used it stated that it should always be well cooked and never eaten raw. Its use was mainly due to the high competition among the population in the search for and collection of *A. caesarea* complex. In contrast to these results, in other states in Mexico a lot of wild species are consumed on a regular basis, for example, in the villages around the volcano La Malinche, Tlaxcala, people regularly consume 74 different mushrooms, and 73 are regularly consumed in Michoacan state. Mestizos of Ozumba in the state of Mexico consume 89 species, the Nahuas of Tlaxcala 66, the mestizos of Federal District 60, the mestizos of the state of Mexico 56, the Purepecha of Michoacan 56, the Ixtlan Zapotec of Oaxaca 33, and the Nahua of Puebla 28 [49]. In total, in the country there are more than 350 species of wild edible fungi [50].

**Table 4:** Common designations by mestizos and Raramuris of some wild edible mushrooms of the Sierra Tarahumara (*Raramuri Language; **Nahuatl Name; ^**1**^ **Local Names)**

Specie	Common allocations
*Amanita caesarea* complex	*Morochike, *Morochic, *Morochiki, *Wicowi., ^1^Amarillo (Yellow), ^1^Árbol del hongo (Tree fungus), ^1^Faldita amarilla (Yellow skirt), ^1^Vestidito amarillo (Yellow dress), ^1^Hongo del agua (Water fungus)
*Amanita rubescens*	*Sojáchic, *Sojáchi, *Serochi, *Sokowekeri, ^1^Hongo del agua (Water fungus)
*Hypomyces lactifluorum*	^1^Trompa de cochi (Pig trunk), *Sokowekeri, ^1^Trompa (Horn)
*Russula brevipes*	*Repome, *Repomi, ^1^Bajío (Shallows), ^1^Semita (semite)
*Laccaria laccata*	*Longongo
*Boletus pinophilus*	*Serochako, ^1^Esponja (Sponge), ^1^Gorro del padre (Father’s bonnet)
*Boletus edulis*	*Serochako, ^1^Esponja (Sponge), *Sonaka, ^1^Gorro del padre (Father’s bonnet), ^1^Panadero (Baker)
*Auricularia polytricha*	^1^Orejona (Big ears)
*Ramaria* aff. *flava*	^1^Cola de vaca (Tail of cow)
*Hericium erinaceus*	*Cha’merówa
*Lactarius indigo*	*Cuauhmiqui
*Agaricus campestris*	^1^Champiñón (Champignon), ^1^Hongo del prado (Fungi of lowland), ^1^Hongo del llano (Fungi of grass), ^1^Del monte (Of mount), *Wecowique, ^1^Llanero (Ranger), *Wecowi
*Schizophyllum commune*	^1^Hongo de la madera (Fungi of the timber), *Amuri, *Pim de amuri
*Ustilago maydis*	**Huitlacoche, Hongo del maíz (Corn’s fungi), *witachori
*Amanita muscaria*	*Guerechaka, *Gerechaka, ^1^Hongo malo (Bad fungi), ^1^rojo (red)
*Amanita virosa*	*Kokohurcobi, ^1^Ángel malo (Bad angel), ^1^Ángel venenoso (Poisonous angel)

### Culinary information and recognition criteria of edible species

*Amanita rubescens*, known as “Sojachi,” is consumed after the cuticle of the pileus is removed and is then washed and cooked with tomato, onion, and garlic. *Hypomyces lactifluorum* (“cochi Trunk”) usually has a lot of dirt on it, and should be cleaned and washed several times. *Amanita caesarea* complex (called “Morochike” or “yellow skirt” or just “yellow”) is cut into pieces and cooked with meat or vegetables and typical spices of Mexico like: chili, *tortillas, corn, beans, and *nopal or as part of common dishes like *pozole (*common names of Mexican foods) or cooked with lard (animal fat) and accompanied by beans. Some people from San Juanito, Arareco and San Rafael claim to wash them in hot water and leave them soaking in water to remove any “hazard” they might carry. They cooked them with garlic in order to test whether they are toxic, using as an indicator a change to black color, indicating that they are poisonous and should not be eaten. This is a common practice throughout the country [50]. However this is not a safe practice as many poisonous mushrooms will react one way or another with the garlic. Also, they admitted that many of the fungi shown in photographs are considered edible elsewhere but they prefer not to eat them for fear, and because they were traditionally considered as bad options. This is a common phenomenon in respect to the utilization of fungi. Moreno Fuentes [25] reported that in another area of the Sierra Tarahumara, the Raramuri did not consume the different species of the genera *Boletus*, *Lactarius,* or *Russula*, which are widely and frequently consumed elsewhere in the country. They prefer to consume only Morochike, Sojachi, and Llanero. In the Arareco area, two people mentioned that they use *Pleurotus* aff. *ostreatus* as food and call it Floera, “Amuri” or “Amuri Pin”. Some people eat *Ramaria* and they recognize *R.* aff. *flava* and call it “cow tail” (Table 4).

### Recorded other uses

Some housewives of San Juanito, mentioned the use of *Amanita caesarea* complex, *H. lactifluorum,* and *A. muscaria* as home decorations and mentioned using dried *Helvella crispa* to make necklaces. Some people of Pitorreal mentioned that *Laccaria laccata* is used medicinally but without specifying the practical use (Table 4). One relevant comment regarding alternative use was for a species of the genus *Lycoperdon* used by a Raramuri of El Divisadero for the medicinal purpose of removing skin warts by placing the inner opening of the fungus on the face. Another farmer mentioned that he used *Boletus edulis* and *B. pinophilus* as food in breeding rams. This use as a forage species is not very common. Ruan-Soto [49] reported the same use of *Russula sp*. for feeding sheep in the highlands of Chiapas in southern Mexico.

### Storage of edible fungi

**Figure 6:**
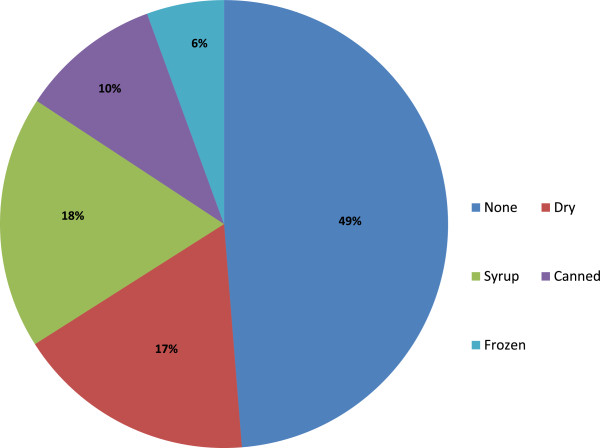
**Storage of edible fungi.**

Forty nine percent of the interviewed (including Raramuris) said that they do not store the mushrooms— they are usually consumed immediately after collecting or buying them; 17% reported that they stored the mushrooms dry; 18% made syrup out of them; and 6% and 10% froze or canned them, respectively (Figure 6). Unlike Mestizos of the Sierra Tarahumara, Raramuris use the term “pass,” which means dehydrating or drying in soil naturally [25]. In general, preservation techniques are not widespread in the rest of the country, although there are some examples of these practices [44] in the center of the country.

### Ways of obtaining fungi

**Figure 7:**
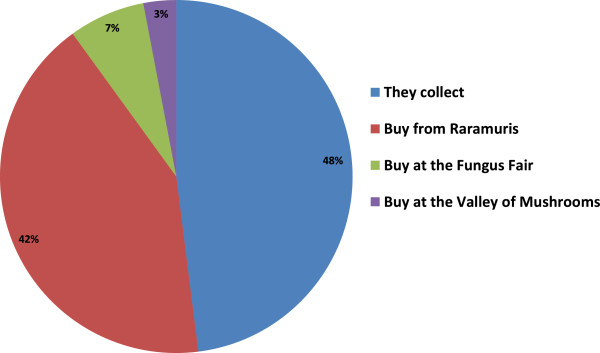
**Ways of obtaining fungi.**

Forty eight percent collect mushrooms directly from the field or forest areas. Forty two percent of the population surveyed reported that they buy their mushrooms from the Raramuris who sell them on the road sides, but sometimes they buy them at their home as a result of door to door to selling. Seven percent mentioned that “Fungus Fair,” which is carried out every year in the month of August provides them with a good opportunity to buy mushrooms and reassures them they are edible (Figure 7). This Fair is an event that has been taking place since 1999 during the first week of August in the town of San Juanito, in the municipality of Bocoyna, with the purpose of spreading [21, 51] the richness of the edible fungal species that grow in the forest areas surrounding the town through means of conferences, gastronomical contests, fungal picking trips, and exhibition of different species. Three percent mentioned that besides selling, people teach other people how to handle mushrooms in the place known as The Valley of the Mushrooms near Arareco Lake [52].

**Figure 8:**
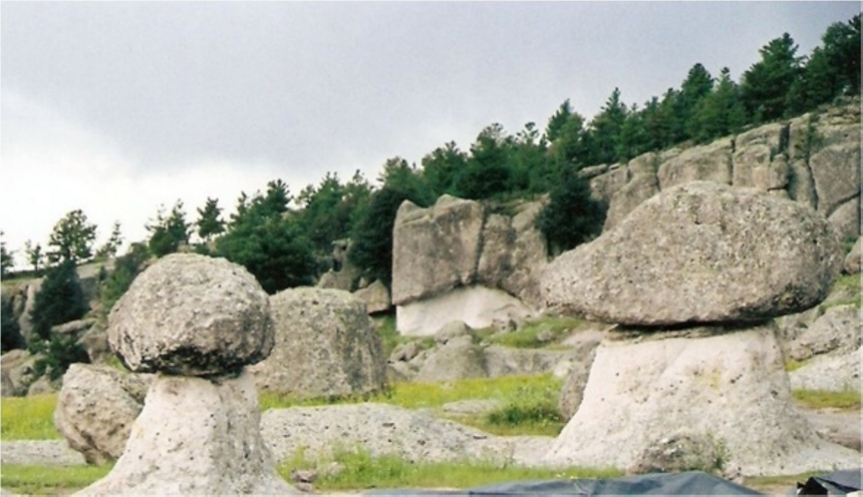
**The valley of the mushrooms.**

The Valley of the Mushrooms is an alpine landscape with rock formations similar to the form of the mushrooms, thus the denomination (Figure 8). People living there are indigenous Raramuris subsisting on agriculture (mainly corn) and, because it is a tourist area, women engage in the selling of handicrafts such as palm baskets of different shapes and woven shawls. In the rainy season, from July to September mainly, they collect and sell Morochike (the common name for *A. caesarea* complex by the people of Bocoyna). It is usually sold with the short stem— that is, they refrain from collecting the volva and the base of the stalk, alleging that they are leaving the “seed” to grow back in the next season.

**Figure 9:**
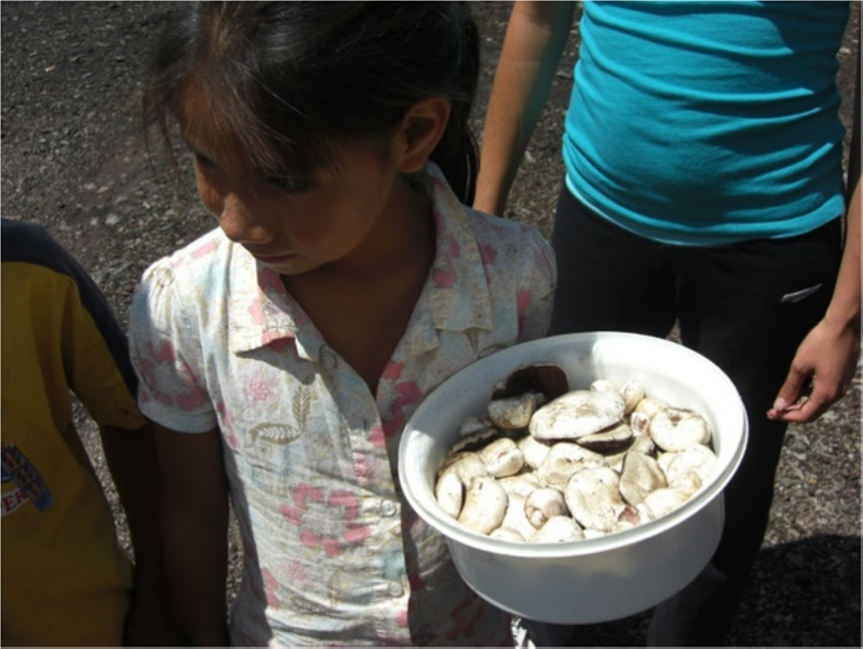
**Children selling** **and** ***Agaricus sp*** **., in July along the road to San Juanito, Chihuahua.**

**Figure 10:**
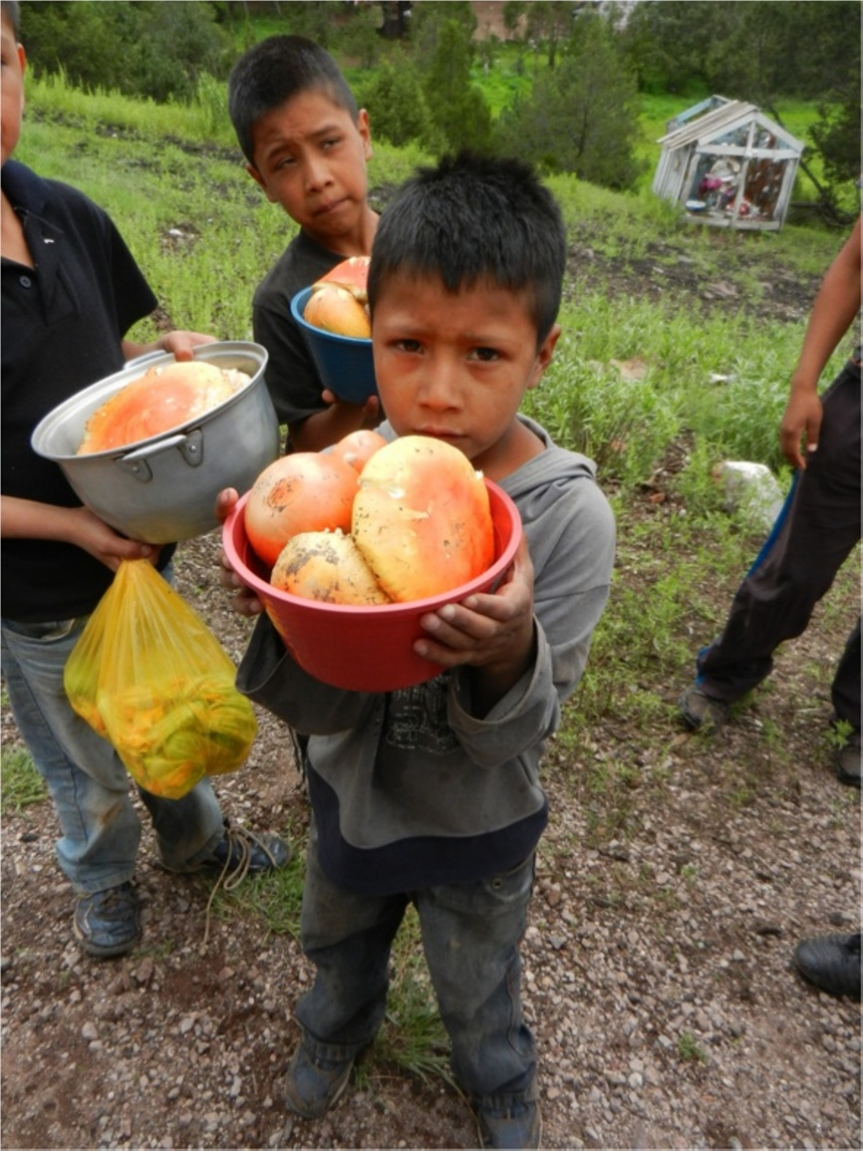
**Children selling** ***Amanita caesarea*** **in the month of August along the road to San Juanito, Chihuahua.**

The results of the interviews, as well as of direct observation were carried out in the forests of the Bocoyna and Urique municipalities; these results showed that in the months of June and July, after the arrival of the first rains, many of the farmers and villagers near San Juanito collect and sell mainly two species: *A. campestris* and *Agaricus sp*. These species are offered along the roads near San Juanito, locally known as mushrooms or wecowi (Figure 9). In August, *A. caesarea* complex and *A. rubescens* are more abundant and are sold on trays or in baskets of different sizes, with the price ranging from $50.00 to $80.00 pesos according to volume (Figure 10), regardless of the species; although, according to the results obtained, *A. caesarea* complex is the most valued species by the people of all the villages studied (Table 3). In other states of Mexico, like in Chiapas in the markets of San Cristobal de Las Casas, at least six species of wild mushrooms, especially *Amanita jaksonii* and *A. hayalyuy*, due to the appreciation that people have for them and taking into account the quantity sold, these can reach up to $50.00 pesos per unit (three or four medium-sized mushrooms) [33].

### Teaching

**Figure 11:**
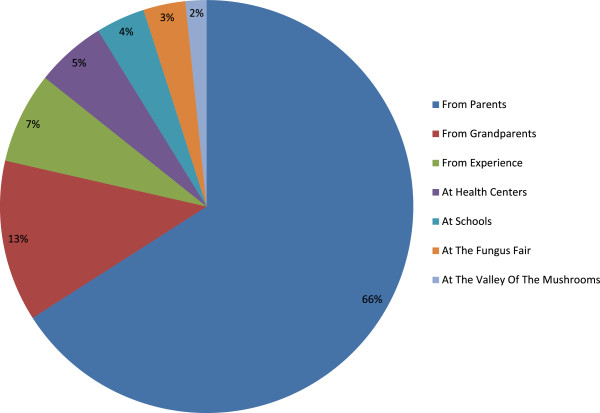
**Teaching obtained knowledge of fungi.**

Sixty-six percent of those interviewed reported that they obtained knowledge of fungi from their parents; 13% from their grandparents; 7% from traditional experience and less than 6% from other sources such as schools, health centers, at a location named “Valley of the Mushrooms” and at the annual Fungus Fair celebrated in San Juanito Chihuahua (Figure 11).

In other states of Mexico, such as Oaxaca and Chiapas, state fairs are organized on mushrooms to teach people and show them that wild mushrooms can be used as safe economic and food alternatives [33].

Based on feedback obtained from the reports of the children interviewed, in primary schools in the communities of Creel and San Juanito, teachers teach students how to distinguish edible mushrooms from poisonous species. This coincides with the studies by Moreno et al. [21] referring to textbooks for the fifth and sixth grades by the Ministry of Education (SEP) about fungi. The main features that people use to differentiate collected fungi are: appearance, color (e.g., red is bad, yellow at the top with white stem is bad, completely yellow is good), grains (flakes) or skirt (ring).

## Conclusions

In the forests of the Sierra Tarahumara, there are records of around 450 species of fungi; 50 of them with edible importance at nationwide and apparently only 16 fungi species of those 50 are being consumed by the inhabitants of the municipalities of Bocoyna and Urique with *Amanita caesarea* complex being the most preferred by mestizos and Raramuris. We observed no apparent differences in the population studied in terms of gender, occupation, or language, regarding the recognition and consumption of species; however, this is not conclusive and so it is important to continue with a greater number of such studies to check whether this knowledge and use is differential.

There is no evidence that shows a meaningful comparison in terms of preferences for wild mushrooms, except for the naming of fungal species.

Many species considered as potentially edible in many regions of Mexico and around the world, such as *Boletus edulis* and *Cantharellus cibarius,* are not recognized as such by the mountain people of Chihuahua. There is a remarkable contrast between the high diversity of wildlife and the low use of species;, while the population knows and appreciates three species in particular, they lack this attention for more than 20 species considered edible in most of Mexico. There is a possibility that they fear poisoning due to some casualties that occurred in the past before the Fungus Fair was established. This event was proposed specifically to generate knowledge in these locations on edible mushroom species. Likewise, there are species that might be used for medicinal purposes but there is no formal study on those used by the Raramuri people for healing; we therefore recommend specific future studies for these purposes.

Finally, in Chihuahua, ethno-mycological development depends largely on two factors: 1) Dissemination of knowledge to villagers, including mestizos and Raramuris, regarding differentiation, the appropriate use of edible wild species growing in the forests of the Sierra Tarahumara, and 2) joining together with professionals, authorities, and the community at large in the conservation on forest resources and thus promoting a more stable environment for the development of wild mushrooms.

## Electronic supplementary material

Additional file 1: Annex: Interview format. (DOCX 15 KB)
