# Supplementary material for: Knowledge and use of edible mushrooms in two municipalities of the Sierra Tarahumara, Chihuahua, Mexico
Source: J Ethnobiol Ethnomed. 2014 Sep 17;10:67. doi: 10.1186/1746-4269-10-67 (PMC4177763; doi:10.1186/1746-4269-10-67)
Supplement: Supplementary file 1 — Additional file 1: Annex: Interview format. (DOCX 15 KB) [file 13002_2013_433_MOESM1_ESM.docx]

**Additional file 1: Annex Interview Format**

Personal data

Name: _________________________________________________ Gender: M/F

Locality: _________________________________ Speaks Raramuri: No/Yes

Age: ______ Studies: _________________ Profession or work: ________________

1. Which of these mushrooms do you know? What name do you give to each of the

fungi?

| **1 ⃝** | **9 ⃝** | **17 ⃝** |
| --- | --- | --- |
| **2 ⃝** | **10 ⃝** | **18 ⃝** |
| **3 ⃝** | **11 ⃝** | **19 ⃝** |
| **4 ⃝** | **12 ⃝** | **20 ⃝** |
| **5 ⃝** | **13 ⃝** | **21 ⃝** |
| **6 ⃝** | **14 ⃝** | **22 ⃝** |
| **7 ⃝** | **15 ⃝** | **23 ⃝** |
| **8 ⃝** | **16 ⃝** | **24 ⃝** |

2. Which of these mushrooms have you eaten and how does it taste?

| **1 ⃝** | **9 ⃝** | **17 ⃝** |
| --- | --- | --- |
| **2 ⃝** | **10 ⃝** | **18 ⃝** |
| **3 ⃝** | **11 ⃝** | **19 ⃝** |
| **4 ⃝** | **12 ⃝** | **20 ⃝** |
| **5 ⃝** | **13 ⃝** | **21 ⃝** |
| **6 ⃝** | **14 ⃝** | **22 ⃝** |
| **7 ⃝** | **15 ⃝** | **23 ⃝** |
| **8 ⃝** | **16 ⃝** | **24 ⃝** |

3. How do you preserve your mushrooms?

4. Which of these fungi do you know that you can eat even though you do not?

5. How do you identify an edible mushroom?

6. Which of these fungi are bad and what are they called?

| **1 ⃝** | **9 ⃝** | **17 ⃝** |
| --- | --- | --- |
| **2 ⃝** | **10 ⃝** | **18 ⃝** |
| **3 ⃝** | **11 ⃝** | **19 ⃝** |
| **4 ⃝** | **12 ⃝** | **20 ⃝** |
| **5 ⃝** | **13 ⃝** | **21 ⃝** |
| **6 ⃝** | **14 ⃝** | **22 ⃝** |
| **7 ⃝** | **15 ⃝** | **23 ⃝** |
| **8 ⃝** | **16 ⃝** | **24 ⃝** |

7. Do you know of another fungus that is not in these pictures? (use and name)

8. Do you know where they are sold and who sells these mushrooms?

9. Where did you learn about mushrooms? Who taught you about mushrooms?

Observations:
